# Anti-colon cancer effect of caffeic acid *p*-nitro-phenethyl ester *in vitro* and *in vivo* and detection of its metabolites

**DOI:** 10.1038/s41598-017-07953-8

**Published:** 2017-08-08

**Authors:** Hao Tang, Xiaofang Yao, Cong Yao, Xiaoyan Zhao, Hua Zuo, Zhubo Li

**Affiliations:** College of Pharmaceutical Sciences, Southwest University, Chongqing, 400716 China

## Abstract

Caffeic acid phenethyl ester (CAPE), extracted from *propolis*, was proven to inhibit colon cancer. Caffeic acid *p*-nitro-phenethyl ester (CAPE-*p*NO_2_), a derivative of CAPE, was determined to be an anti-platelet agent and a protector of myocardial ischaemia with more potent effects. In the present study, CAPE-*p*NO_2_ showed stronger cytotoxic activity than CAPE. We revealed interactions between CAPE-*p*NO_2_ and experimental cells. CAPE-*p*NO_2_ induced apoptosis in HT-29 cells by up-regulating P53, cleaved-caspase-3, Bax, P38 and CytoC; CAPE-*p*NO_2_ also up-regulated P21^Cip1^ and P27^Kip1^ and down-regulated CDK2 and c-Myc to promote cell cycle arrest in G0/G1. In xenograft studies, CAPE-*p*NO_2_ remarkably suppressed tumour growth dose dependently and decreased the expression of VEGF (vascular endothelial growth factor) in tumour tissue. Moreover, HE staining showed that no observable toxicity was found in the heart, liver, kidney and spleen. In addition, metabolites of CAPE-*p*NO_2_ in HT-29 cells and organs were detected. In conclusion, para-nitro may enhance the anticancer effect of CAPE by inhibiting colon cancer cell viability, inducing apoptosis and cell cycle arrest via the P53 pathway and inhibiting tumour growth and reducing tumour invasion by decreasing the expression of VEGF; additionally, metabolites of CAPE-*p*NO_2_ showed differences in cells and organs.

## Introduction

Colon cancer is a common malignant tumour of the digestive tract. In clinical settings, approximately 1.4 million colon cancer patients were diagnosed and more than 690 thousand patients died from colon cancer worldwide in 2012^1^. Risk factors of colon cancer mainly include increasing age, male sex, a high-fat diet, an inadequate intake of fibre and a sedentary lifestyle. Nearly 50% of patients die from recrudescence and metastatic diseases two years after curative resections^2, 3^. Based on a series of statistical data, lower expression of Bcl-2 and overexpression of Bax contribute to the lower survival rate in colon cancer patients. Caspase-9 overexpression leads to cell apoptosis and G0/G1 arrest and reduces the secretion of carcinoembryonic antigen, and Caspase-3 can induce the damage of DNA^4–7^. The abnormal expression of cell cycle-associated proteins (c-Myc and CDK2) may promote the occurrence of colon cancer^8–11^. Wang, J. J. *et al*. reported that 1, 6-bis [4-(4-amino-3-hydroxyphenoxy) -phenyl] (DPD) inhibited colon cancer cell (HCT-116) activity and tumour growth through the P21 signalling pathway^12^. These studies implied that the occurrence of colon cancer is related to the P53 pathway.

Caffeic acid phenethyl ester (CAPE) has been identified as the main active component of *propolis*. It has been reported that CAPE possesses antioxidant, anti-inflammatory and anti-cancer effects^13–15^. It can inhibit prostate cancer by regulating the expression of Skp2, P27^Kip1^, P21^Cip1^ and P53, reduce VEGF secretion in MDA-231 cells in a dose-dependent manner^16–18^; up-regulate E2F-1, P53 and P21 expression in cervical cancer cells; and change the expression of Cyclin A, Cyclin B and Cyclin C in the cell cycle^19^. Additionally, CAPE exerts therapeutic effects on cholangiocarcinoma, lung cancer, liver cancer and oral cancer^20–23^. In our previous study, caffeic acid para-nitro-phenethyl ester (CAPE-*p*NO_2_) was developed and proven to be more effective than CAPE in acute myocardial ischaemic and reperfusion injury in rats and other blood-related diseases in previous studies^24, 25^. However, after the introduction of the nitro-group in CAPE, the structure-dependent changes in the anticancer activity of the CAPE derivatives against colon cancer are still unknown. Hence, we comparatively studied the anti-colon cancer effects of CAPE-*p*NO_2_ and CAPE, and detected the metabolites by LC-MS/MS in colon cancer cells, xenograft tumour and organs.

## Results

### CAPE and CAPE-*p*NO_2_ inhibit cell proliferation

**Figure 1:**
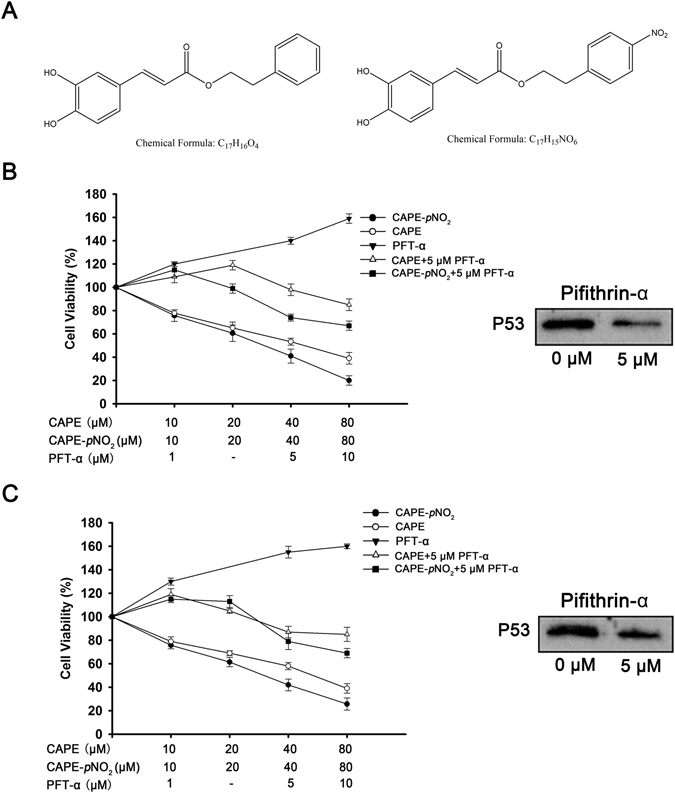
Chemical structure of the compounds used in the present study and results of the MTT assay. (**A)** Chemical structure of CAPE and CAPE-*p*NO_2_. HT-29 cells **(B)** and HCT-116 cells **(C)** were treated with CAPE and CAPE and CAPE-*p*NO_2_ for 48 h, and the expression of P53 after treatment by PFT-α. Values represented the means ± SD from three independent experiments, and error bars represented the STDEV (SD).

The inhibitory effects of CAPE and CAPE-*p*NO_2_ on colon cancer cells were measured by the MTT assay. In HT-29 cells (Fig. 1B), CAPE and CAPE-*p*NO_2_ inhibited cell viability in a dose-dependent manner, the IC_50_ of CAPE and CAPE-*p*NO_2_ were 44.5 μM and 29.7 μM. For HCT-116 cells (Fig. 1C), the IC_50_ of CAPE and CAPE-*p*NO_2_ were 47.2 μM and 33.8 μM. The cell viability of HT-29 cells and HCT-116 cells was increased after PFT-α treating, and PFT-α decreased the effect of CAPE and CAPE-*p*NO_2_.

### CAPE and CAPE-*p*NO_2_ induce colon cancer cell apoptosis

**Figure 2:**
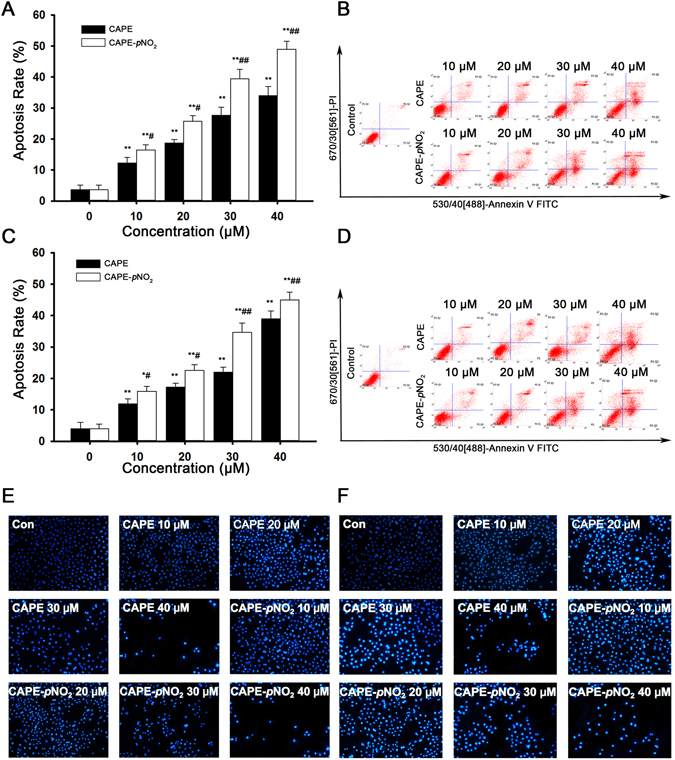
CAPE and CAPE-*p*NO_2_ induce apoptosis in colon cancer cells. HT-29 and HCT-116 cells were treated with 0, 10, 20, 30 and 40 μmol/L CAPE and CAPE-*p*NO_2_ for 48 h. The apoptosis rates of HT-29 cells **(A)** and HCT-116 cells **(B)** were calculated by SigmaPlot 12.5. Flow cytometry analysis in HT-29 **(C)** and HCT-116 **(D)** cells. HT-29 cells **(E)** and HCT-116 **(F)** cells (×200) were stained by Hoechst 33342 after treatment with different concentrations of CAPE and CAPE-*p*NO_2_ for 48 h. From the microscopic vision fields, the cell number was decreased, and fluorescence was increased by treatment for 48 h in a dose-dependent manner. Values represented the means ± SD from three independent experiments, and error bars represented the STDEV (SD). *p < 0.05, **p < 0.01: CAPE and CAPE-*p*NO_2_ compared with the control. ^#^ *p* < 0.05, ^##^ *p* < 0.01: CAPE-*p*NO_2_ compared with CAPE at the same concentration.

After 48 h of treatment, the apoptosis rates were identified by flow cytometry (Fig. 2C,D). The data showed that CAPE-*p*NO_2_ induced colon cancer cell apoptosis in a dose-dependent manner, and the effect of CAPE-*p*NO_2_ was distinctly higher than that of CAPE. At 40 μM, the apoptosis rates in the CAPE and CAPE-*p*NO_2_ treatment groups were 34.0% and 49.0%, respectively, in HT-29 cells and 39.0% and 47.0%, respectively, in HCT-116 cells (Fig. 2A,B). Hoechst 33342 staining was also used to measure cell apoptosis. From the microscopic vision fields, the number of HT-29 and HCT-116 cells was decreased, and the fluorescence intensity was increased with increasing concentrations of CAPE and CAPE-*p*NO_2_. The results suggested that the effect of CAPE-*p*NO_2_ is stronger than that of CAPE (*p* < 0.01) (Fig. 2E,F).

### CAPE and CAPE-*p*NO_2_ induce cell cycle arrest in G0/G1 in colon cancer cells

**Figure 3:**
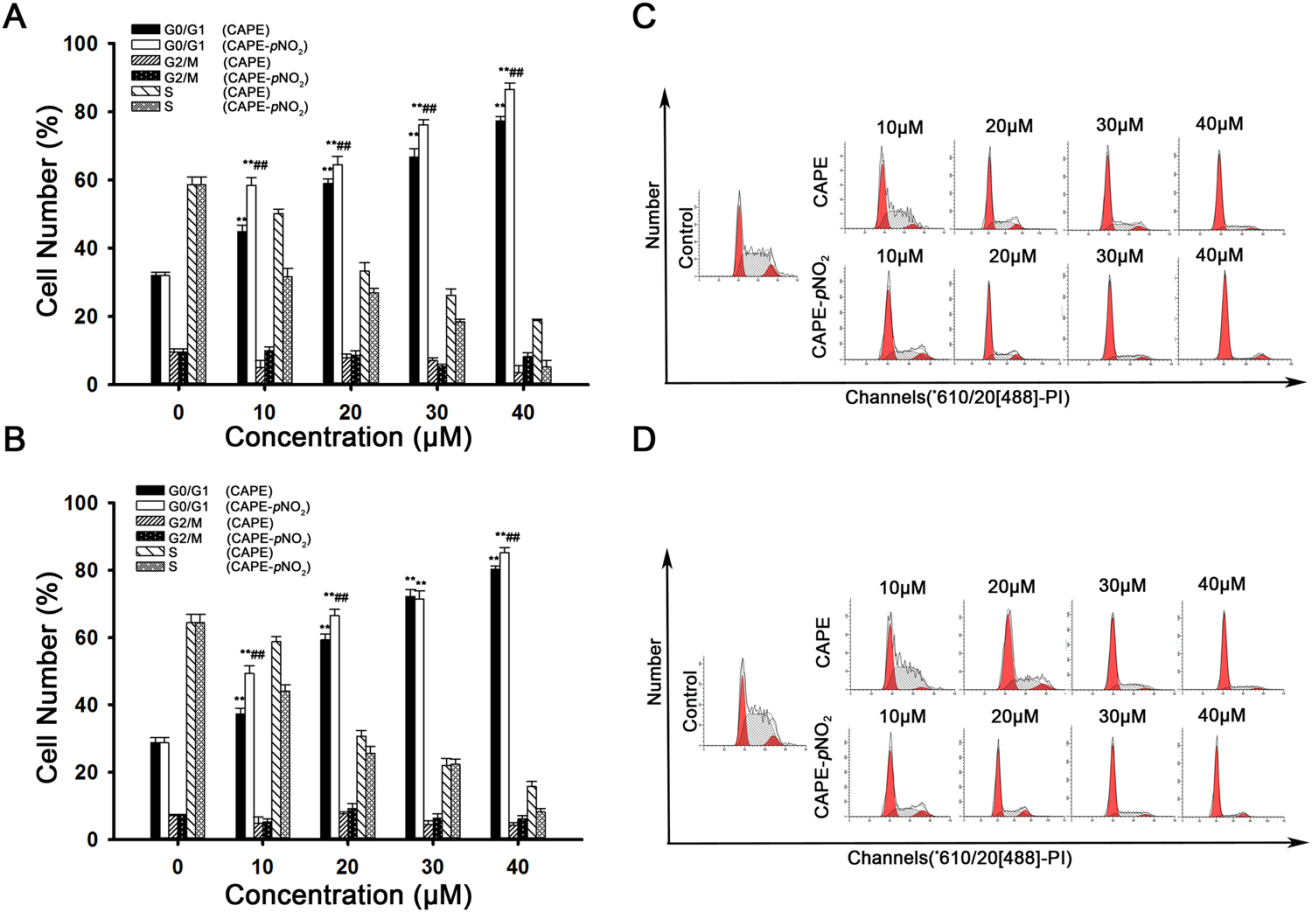
CAPE and CAPE-*p*NO_2_ induce cell cycle arrest at the G0/G1 phase in colon cancer cells. The percentage of cells in G0/G1, S and G2/M phase was calculated using Multicycle software. Changes in the cell cycle distribution in HT-29 **(A)** and HCT-116 **(B)** cells after treatment for 48 h with the concentration from 0 to 40 μmol/L. HT-29 **(C)** and HCT-116 **(D)** cells were stained with PI and were analysed by flow cytometry. The data represented the means ± SD from three independent experiments, and error bars represented with STDEV (SD). *p < 0.05, **p < 0.01: CAPE and CAPE-*p*NO_2_ compared with the control. ^#^ *p* < 0.05, ^##^ *p* < 0.01: CAPE-*p*NO_2_ compared with CAPE at the same concentration.

The cell cycle distribution was detected by flow cytometry. As shown in Fig. 3, the number of HT-29 cells (Fig. 3A) in G0/G1 phase increased in a dose-dependent manner from 31.9% to 77.3% and 86.5% after treatment with CAPE and CAPE-*p*NO_2_, respectively. Concerning HCT-116 cells (Fig. 3B), the G0/G1 phase increased from 31.0% to 80.1% and 84.5%, but the G2/M phase was barely changed in the present study. The results proved that the progression of cells from G1 to the S phase was interrupted more obviously by CAPE-*p*NO_2_ than by CAPE (*p* < 0.01) (Fig. 3C,D).

### CAPE and CAPE-*p*NO_2_ regulate the expression of P53 signalling pathway related proteins in HT-29 cell and tumours

**Figure 4:**
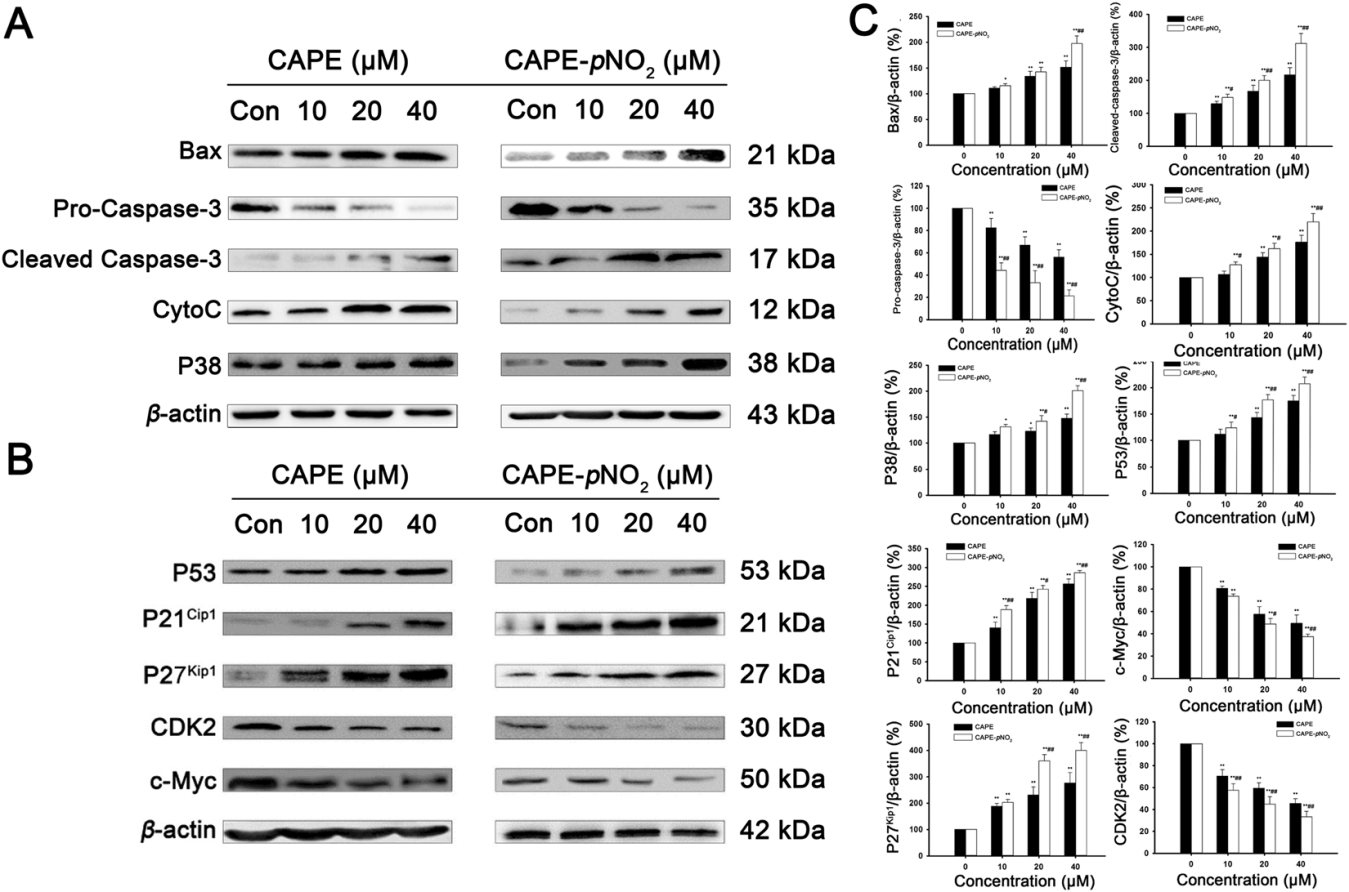
Regulation of apoptosis-related and cycle-related proteins by CAPE and CAPE-*p*NO_2_ treatment in HT-29 cells. HT-29 cells were treated with CAPE and CAPE-*p*NO_2_ at a dose of 0, 10, 20 or 40 μmol/L for 48 h. **(A)** Apoptosis-related proteins in the P53 signalling pathway such as Bax, Pro-Caspase-3, Cleaved-Caspase-3, CytoC and P38 were determined by western blotting. **(B)** The cell cycle-related protein expression levels of P21 ^Cip1^, P27^Kip1^, P53, CDK2 and c-Myc were determined by western blotting. The blots were representative of three independent experiments. Data represented the means ± SD from three independent experiments, and error bars represented the STDEV (SD). *p < 0.05, **p < 0.01: CAPE and CAPE-*p*NO_2_ compared with the control. ^#^ *p* < 0.05, ^##^ *p* < 0.01: CAPE-*p*NO_2_ compared with CAPE at the same concentration.

The expression of related proteins in HT-29 cells was measured by western blot assay after treatment with CAPE and CAPE-*p*NO_2_ (Fig. 4). The results showed that these two drugs could down-regulate the expression of pro-caspase-3, which was reduced by 44.0% and 79.0% after treatment with CAPE and CAPE-*p*NO_2_, respectively, while cleaved-caspase-3 was increased 2.1- and 3.1-fold, respectively. CAPE and CAPE-*p*NO_2_ could up-regulate the expression of Bax, CytoC, P53 and P38, and the up-regulated specific data were 0.5- and 0.9-fold in Bax, 0.7- and 1.1-fold in CytoC, 0.7- and 1.0-fold in P53, and 0.43- and 0.78-fold in P38, respectively. The above effects occurred in a dose-dependent manner within 10 μM to 40 μM. These results showed that CAPE and CAPE-*p*NO_2_ induce HT-29 cell apoptosis likely through the P53 signalling pathway, and the effect of CAPE-*p*NO_2_ is stronger. CAPE and CAPE-*p*NO_2_ also regulated cell cycle-related proteins. After treatment with CAPE and CAPE-*p*NO_2_ at 40 μM for 48 h, C-Myc and CDK2 were reduced by 51.0%, 63.0% and 55.0%, 67.0%, while P21^Cip1^ and P27^kip1^ were increased 1.7- and 1.8-fold and 1.7- and 3.0-fold. In tumours, CAPE-*p*NO_2_ could regulate expression of P53 signaling pathway related proteins in a dose-dependent manner. CAPE and CAPE-*p*NO_2_ could up-regulated P53, P21^Cip1^, P27^Kip1^, CytoC and Cleaved Caspase-3, down-regulated c-Myc, CDK2 and Pro-Caspase-3. After CAPE and CAPE-*p*NO_2_ treating in the same dose of 10 mg/kg/day, the expressions of these proteins were up to 114% and 183% in P53, 180% and 260% in P27^Kip1^, 137% and 207% in P21^Cip1^, 113% and 183% in CytoC, 120% and 206% in Cleaved Caspase-3, while down to 63% and 49% in Pro-Caspase-3, 80% and 51% in CDK2, 82% and 53% in c-Myc, respectively (Fig. 7).

**Figure 5:**
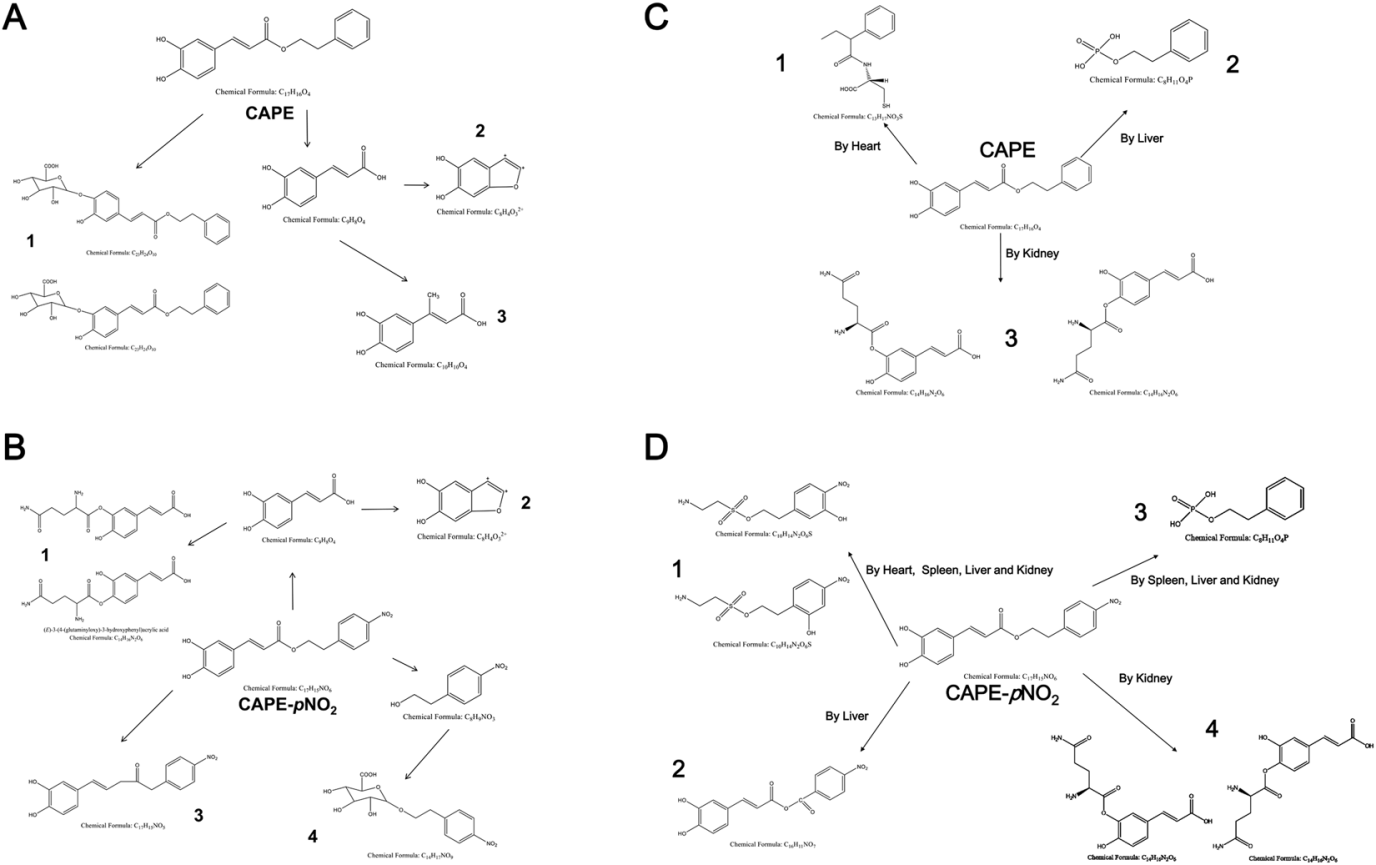
Metabolite analysis of CAPE and CAPE-*p*NO_2_ in HT-29 cells and in tumours, heart, liver, spleen and kidney. Metabolic processes of CAPE **(A)** and CAPE-*p*NO_2_ **(B)** in HT-29 cells. Metabolite analysis of CAPE **(C)** and CAPE-*p*NO_2_ **(D)** in tumours, heart, liver, spleen and kidney.

### Metabolite analysis in HT-29 cells, xenograft tumour and several organs after treating with CAPE and CAPE-*p*NO_2_

The metabolites of CAPE and CAPE-*p*NO_2_ in HT-29 cells were analysed using the Triple TOFTM 4600 system and MetabolitePilot 1.5 software. Three metabolites of CAPE were detected: (2S,3S,4R,5R,6S)-3,4,5-trihydroxy-6-(2-hydroxy-5-((E)-3-oxo-3-phenethoxyprop-1-en-1-yl) phenoxy)tetrahydro-2H-pyran-2-carboxylic acid or (2S,3S,4R,5R,6S)-3,4,5-trihydroxy-6-(2- hydroxy-4-((E)-3-oxo-3-phenethoxyprop-1-en-1-yl)phenoxy)tetrahydro-2H-pyran-2-carboxylic acid **(C**_**23**_ **H**_**24**_ **O**_**10**_ **)**, 5,6-dihydroxybenzofuran-2,3-diylium **(C**_**8**_ **H**_**4**_ **0**_**3**_^**2+**^ **)** and (E)-3-(3,4- dihydroxyphenyl) but-2-enoic acid **(C**_**10**_ **H**_**10**_ **O**_**4**_ **)**. Four metabolites of **CAPE-** ***p*** **NO**_**2**_ were detected: (E)-3-(4-(glutaminyloxy)-3-hydroxyphenyl) acrylic acid or (E)-3-(3- (glutaminyloxy)-4-hydroxyphenyl) acrylic acid **(C**_**10**_ **H**_**10**_ **O**_**4**_ **)**, 5,6-dihydroxybenzo-furan-2,3 -diylium **(C**_**8**_ **H**_**4**_ **0**_**3**_^**2+**^ **)**, (E)-5-(3,4-dihydroxyphenyl)-1-(4-nitrophenyl) pent-4-en-2-one **(C**_**17**_ **H**_**15**_ **NO**_**5**_ **)** and (2S,3S,4R,5R)-3,4,5-trihydroxy-6-(4-nitrophenethoxy)tetrahydro-2H -pyran -2-carboxylic acid **(C**_**14**_ **H**_**17**_ **NO**_**9**_ **)**. Only C_8_H_4_0_3_^2+^ was a common metabolite of CAPE and CAPE-*p*NO_2_ (Figs 5A,B and S1).

**Figure 6:**
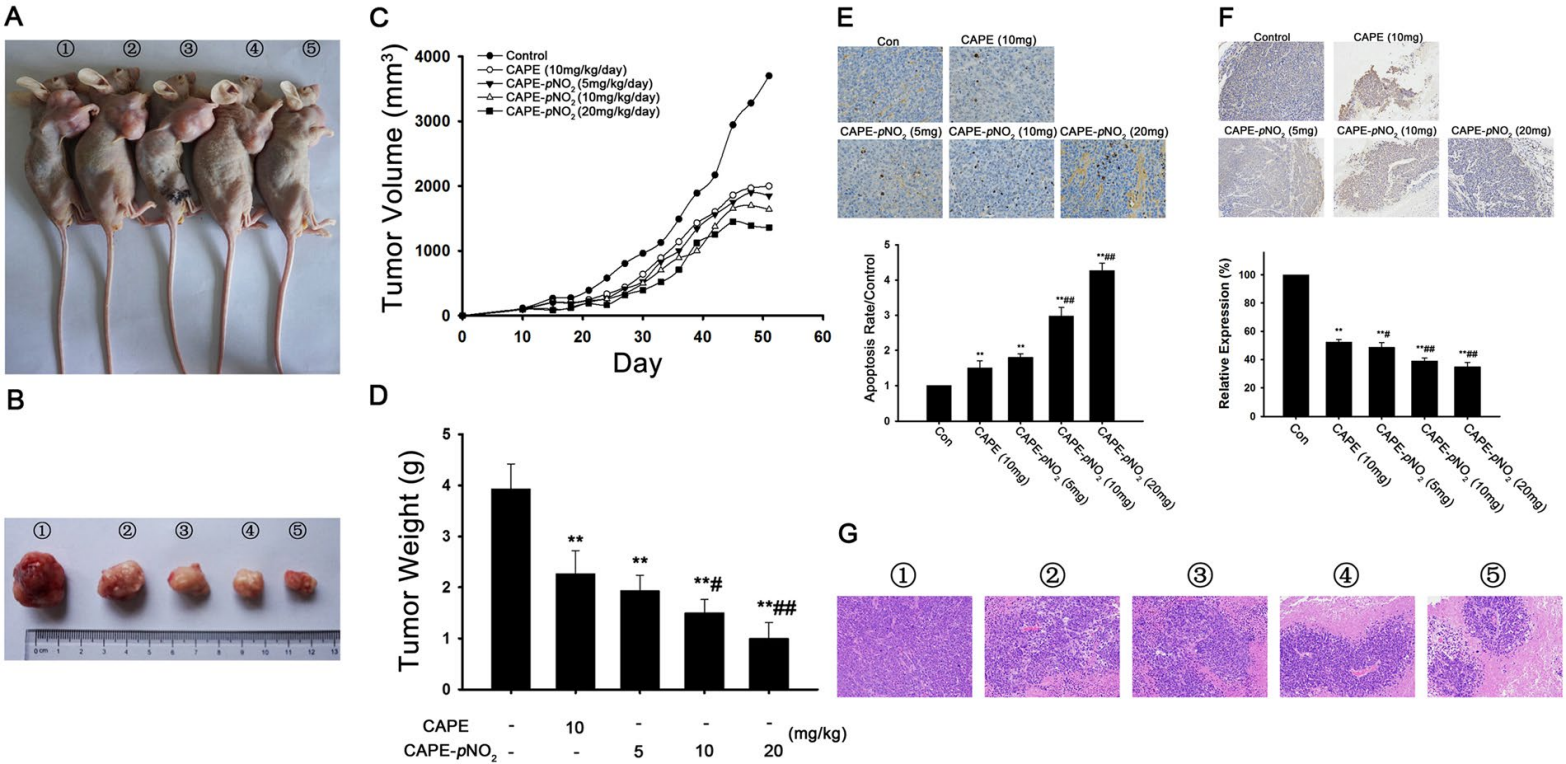
CAPE and CAPE-*p*NO_2_ inhibit tumour growth *in vivo*. After 41-day treatment, tumours were observed *in vivo* **(A)** and *in vitro* **(B)**; ①, ②, ③, ④ and ⑤ represented the control, CAPE-10 mg/kg/day, CAPE-*p*NO_2_-5 mg/kg/day, CAPE-*p*NO_2_-10 mg/kg/day and CAPE-*p*NO_2_-20 mg/kg/day groups, respectively. **(C)** After nude mice were injected with HT-29 cells for 9 days, CAPE and CAPE-*p*NO_2_ were given intragastrically for 42 days. The tumour volume was measured every three days. **(D)** Inhibition rate of tumour growth after treatment with CAPE and CAPE-*p*NO_2_. Apoptosis and expression were detected by TUNEL and immunohistochemistry. **(E)** TUNEL staining in tumours cells (×400), and the relative apoptosis rate was calculated using Image-Pro Plus (IPP) software. **(F)** Results of immunohistochemistry (×200). The integrated option density (IOD) value was used to measure the expression level of VEGF. **(G)** The paraffin sections of tumours were stained with haematoxylin and eosin (HE), and the necrotic area and shrinking nucleus were increased after treatment in a dose-dependent manner; ①, ②, ③, ④ and ⑤ represented the control, CAPE-10 mg/kg/day, CAPE-*p*NO_2_-5 mg/kg/day, CAPE-*p*NO_2_-10 mg/kg/day and CAPE-*p*NO_2_-20 mg/kg/day groups. Values represented the means ± SD from three independent experiments, and error bars represented the STDEV (SD). *p < 0.05, **p < 0.01: CAPE and CAPE-*p*NO_2_ compared with the control. ^#^ *p* < 0.05, ^##^ *p* < 0.01: CAPE-*p*NO_2_ (5, 10, 20 mg/kg/day) compared with CAPE (10 mg/kg/day).

CAPE was transformed into (2-phenylbutanoyl)-D-cysteine (**C**_**13**_ **H**_**17**_ **NO**_**3**_ **S**) in the heart, phenethyl dihydrogen phosphate (**C**_**8**_ **H**_**11**_ **O**_**4**_ **P**) in the liver, and (E)-3-(3-((L-glutaminyl) oxy)-4-hydroxyphenyl) acrylic acid or (E)-3-(4-((D-glutaminyl) oxy)-3-hydroxyphenyl) acrylic acid (**C**_**14**_ **H**_**16**_ **N**_**2**_ **O**_**6**_) in the kidney. However, CAPE-*p*NO_2_ was metabolized into 3-hydroxy-4-nitrophenethyl 2-aminoethane-1-sulfonate or 2-hydroxy-4-nitrophenethyl 2-aminoethane-1-sulfonate (**C**_**10**_ **H**_**14**_ **N**_**2**_ **O**_**6**_ **S**) in the heart, liver, spleen and kidney and phenethyl dihydrogen phosphate (**C**_**8**_ **H**_**11**_ **O**_**4**_ **P**) in the liver, spleen and kidney. Additionally, (E)-(E)-3-(3,4-dihydroxyphenyl) acrylic 4-nitrobenzoic anhydride (**C**_**16**_ **H**_**11**_ **NO**_**7**_) and (E)-3-(3-((L-glutaminyl) oxy)-4-hydroxyphenyl) acrylic acid or (E)-3-(4-((D-glutaminyl) oxy) -3-hydroxyphenyl) acrylic acid (**C**_**14**_ **H**_**16**_ **N**_**2**_ **O**_**6**_) were found in the liver and kidney, respectively (Figs 5C,D and S2,S3). Notably, all of the above metabolites were not found in xenograft tumours.

### CAPE and CAPE-*p*NO_2_ inhibit tumour growth in nude mice

HT-29 cells were xenografted into the nude mice to evaluate the effects of CAPE and CAPE-*p*NO_2_ on tumour growth. As shown in Fig. 6, following the administration of CAPE and CAPE-*p*NO_2_ by gavage for 42 days, tumour growth in the CAPE and CAPE-*p*NO_2_ groups was inhibited remarkably. Compared with the control, the tumour growth inhibition ratios in the CAPE and CAPE-*p*NO_2_ groups (5 mg/kg/day, 10 mg/kg/day, 20 mg/kg/day) were 45.0%, 50.0%, 55.7% and 63.2%, respectively (Fig. 6D). As shown in Fig. 6B, the sizes of tumours after treatment with these two substances were significantly smaller than those of the control group. The results of the tumour and organs by HE staining is shown in Fig. 6G (tumour) and Fig. S4 (heart, liver, spleen and kidney), and they showed that the necrotic area of tumours was expanded after treatment with CAPE and CAPE-*p*NO_2_, but there were no morphological changes in other organs, indicating that CAPE and CAPE-*p*NO_2_ had no visible toxicity. The results of the TUNEL assay in tumours showed that the dose-dependent effects of cell apoptosis induced by CAPE-*p*NO_2_ are stronger than those induced by CAPE (Fig. 6E). Furthermore, immunohistochemistry assay indicated that the expression of VEGF was reduced by 44.0%, 49.0%, 58.0% and 62.0%, respectively, after treatment with CAPE (10 mg/kg) and CAPE-*p*NO_2_ (5 mg/kg, 10 mg/kg and 20 mg/kg) (Fig. 6F).

**Figure 7:**
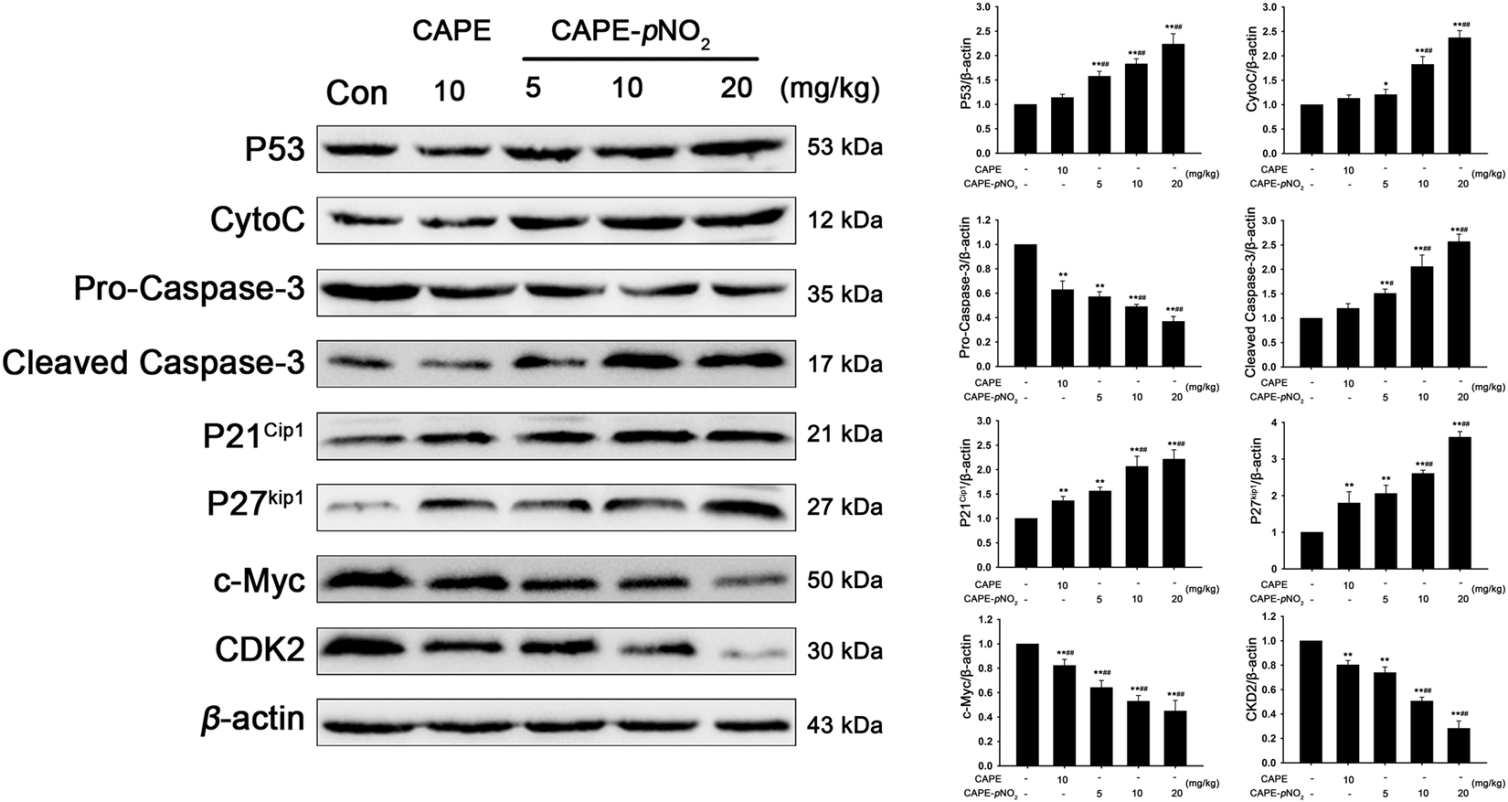
Regulation of proteins by CAPE and CAPE-*p*NO_2_ treatment in in tumours. The regulations of P53, Pro-Caspase-3, Cleaved Caspase-3, CytoC, P21^Cip1^, P21^Kip1^, c-Myc and CDK2 in tumours were detected by western blotting. The blots were representative of three independent experiments. Data represented the means ± SD from three independent experiments, and error bars represented the STDEV (SD). *p < 0.05, **p < 0.01: CAPE and CAPE-*p*NO_2_ compared with the control. ^#^ *p* < 0.05, ^##^ *p* < 0.01: CAPE-*p*NO_2_ compared with CAPE at the same concentration.

## Discussion

CAPE is a bioactive natural ingredient exacted from *propolis* and exhibits inhibition activity against various cancers, such as prostate cancer, breast cancer and colon cancer. In a previous study, the inhibitory effect of CAPE on colon cancer cells was determined. Wang D. *et al*.^26^ reported that CAPE induced HCT-116 cell cycle arrest in G0/G1 and apoptosis by decreasing the expression of β-catenin. Xiang D. *et al*.^27^ suggested that CAPE inhibited the proliferation of HCT-116 cells and SW480 cells via the β-catenin/T-cell signalling pathway by down-regulating cyclin D1 and c-myc, and En-Pei Isabel Chiang *et al*.^28^ discovered that caffeic acid phenylpropyl ester (CAPPE), a derivative of CAPE, could inhibit the growth of the HCT-116 and SW480 cells more significantly than CAPE through the PI3K/AKT and AMPK signalling pathways *in vivo* and *in vitro*. He, Y. J. *et al*.^3^ also proved that CAPE induced SW480 cell apoptosis by down-regulating PSMA1 and PSAT1 while up-regulating GNPDA1 and GPX-1. These studies proved that the signalling pathway that mediated the apoptosis of colon cancer cells and tumour after treatment with CAPE was not unique. P53 is one of the tumour suppressor proteins that has an intimate connection with the occurrence and progression of many tumours in humans, and it can induce apoptosis and cell cycle arrest of carcinoma^29^. The result in the above literature was not involved in the P53 signalling pathway after treatment with CAPE in colon cancer. By contrast, the P53 signalling pathway was selected to explore the anti-cancer mechanism of CAPE and CAPE-*p*NO_2_ in HT-29 cells.

CAPE-*p*NO_2_, a derivative of CAPE like CAPPE but with quite a different chemical structure, was synthesized by adding a nitro moiety to the para position in our laboratory. It was proven to be a platelet anticoagulant for collagen-induced platelet aggregation and a protector of acute myocardial ischaemia-reperfusion injury in previous studies^24, 25^, and these effects of CAPE-*p*NO_2_ were more potent than those of CAPE, but there is no report concerning the anti-cancer effect of CAPE-*p*NO_2_.

In this study, the MSI-type cell line HCT-116 and the MSS-type cell lines HT-29 and SW480 were selected to verify the anti-cancer effect of CAPE and explore the anti-cancer effect of CAPE-*p*NO_2_ in colon cancer. In the MTT assay, CAPE and CAPE-*p*NO_2_ inhibited cell proliferation in a dose-dependent manner, and the IC_50_ of CAPE-*p*NO_2_ was lower than that of CAPE. Moreover, the inhibitory effect of CAPE and CAPE-*p*NO_2_ was weaken when the expression of P53 was decreased by PFT-α, this result proved that P53 signalling pathway was one of the approaches which inhibited the proliferation of HT-29 and HCT-116 cells. Hoechst 33342 staining showed that the number of cancer cell was decreased, and the fluorescence intensity was increased with increasing concentrations of drugs. Compared with the CAPE treated group, after treated with CAPE-*p*NO_2_, the apoptosis rate of HT-29 cell and HCT-116 cells increased 15 and 8 percentage points (Fig. 2), the number of HT-29 cells and HCT-116 cells in G0/G1 phase were increased 10 and 5 percentage points (Fig. 3), these data showed that CAPE-*p*NO_2_ exhibited stronger inhibitory effect on HT-29 cells than that on HCT-116 cells. Based on the above results, we selected HT-29 cells of MSS type cell lines for the further study on anti-colon cancer mechanism of CAPE-*p*NO_2_ *in vivo* and *in vitro*.

The results of western blotting showed that both CAPE and CAPE-*p*NO_2_ could up-regulate P53, P38, Bax, CytoC and cleaved-caspase-3 and down-regulate pro-caspase-3. These proteins associated with the P53 signalling pathway were closely connected to the occurrence and growth of tumours^30^, and up-regulated CytoC proved that CAPE and CAPE-*p*NO_2_ could induce the mitochondrial apoptotic and active pro-caspase-3 to become cleaved-caspase-3, our results were consistent with those of Liu X. *et al*.^31^.

In a study of the cell cycle, CAPE and CAPE-*p*NO_2_ induced colon cancer cell cycle arrest in the G0/G1 phase; up-regulated P53, P21^Cip1^and P27^Kip1^; and down-regulated CDK2 and c-Myc. P53 mainly promoted tumour cell apoptosis and induced cell cycle arrest. When the cells of the body are damaged, P21^Cip1^ mRNA and protein expression levels are elevated following activation by P53 protein, and the cell cycle is blocked in G1, G2 or S phase^32, 33^. Down-regulated CDK2 by CAPE and CAPE-*p*NO_2_ treatment might induce pRb dephosphorylation to promote cancer cell ageing and prevent cell cycle progression from G1 to S phase^34, 35^. Up-regulated c-Myc induces cell cycle arrest in the G1 phase and inhibits the repair effects on telomeres, preventing the cells from being immortalized^36, 37^. Gao FH. *et al*. reported^38^ that oridonin can suppress colon cancer effectively by regulating the expression of c-Myc, P21^Cip1^ and P27^Kip1^. Compared with CAPE, CAPE-*p*NO_2_ more strongly induces up-regulation of Bax, cleaved-caspase-3, CytoC, P53, P38, P21^Cip1^ and P27^Kip1^ and down-regulation of pro-caspase-3, CDK2 and c-Myc. In one word, CAPE and CAPE-*p*NO_2_ inhibited proliferation of cells and suppressed tumours growth by regulating the P53 signalling pathway, and CAPE-*p*NO_2_ is more effective than CAPE in inhibiting cell growth, inducing apoptosis and cell cycle arrest in G0/G1 and suppressed tumours growth.

To explore the anticancer effect of CAPE-*p*NO_2_ *in vivo*, HT-29 cells were xenografted into nude mice. The turning point of the tumour growth curve appeared on the 37th, 35th and 33rd days after treating with CAPE-*p*NO_2_ at doses of 5, 10 and 20 mg/kg/day, respectively. However, the tumour growth curve in the CAPE (10 mg/kg/day) group showed a relatively steady trend on the 41st day. Based on the results *in vitro*, we used HT-29 cells to establish xenograft models. According to the report by En-Pei Isabel Chiang^28^, after injecting HCT-116 cells into nude mice, CAPE treatment lasted for six weeks. Although CAPE markedly inhibited the tumour growth, the growth trend of the treatment group showed a downward trend, a finding that was different from ours. The cause of this discrepancy might be the different cell lines used. Additionally, Wu J^20^ reported that the growth curves of xenograft tumours using MDA-231 cells and MCF-7 cells after treatment with CAPE were also different. At the end of experiment, all nude mice were euthanized, and the tumours were removed. The results of HE and TUNEL staining showed that CAPE and CAPE-*p*NO_2_ inhibited tumour growth through inducing tumour tissue necrosis and apoptosis. More importantly, CAPE-*p*NO_2_ exhibited more potent effects than CAPE on tumours. Interestingly, no morphological changes were found in the heart, liver, spleen and kidney after treatment with CAPE and CAPE-*p*NO_2_ for a long time. In clinical settings, many drugs used to cure colorectal cancer, such as 5-fluorouracil, have serious toxic and side effects and can even lead to patient death^39, 40^. Thus, CAPE-*p*NO_2_ might have great clinical application value. Meanwhile, the results of immunohistochemistry indicated that CAPE and CAPE-*p*NO_2_ decreased the expression of VEGF to disturb the pervasion and growth of tumours, while there was almost no expression of VEGF in normal colon tissues, and many reports have shown that the combination of VEGF with tyrosine kinases and neuropilins on the tumour cell surface promoted the progress of tumour invasion and cancer stem cell formation^41–43^, and VEGF could be related to the survival of patients with colorectal carcinoma and should be considered a predictor of the prognosis clinically^44^. Thus, CAPE-*p*NO_2_ may be regarded as a better inhibitor of VEGF in colon tumours (*p* < 0.01).

The nitro group at the para position was the only difference between CAPE and CAPE-*p*NO_2_. Consequently, our results imply that the anticancer effects of CAPE were enhanced by the para-nitro moiety. Similarly, it was confirmed that para-nitric oxide-donating acetylsalicylic acid was more purposeful in chronic lymphocytic leukaemia cells and more applicable to clinical treatment than NO-ASA^45^. For further study on the para nitro, LC-MS/MS was applied to investigate the difference in metabolites between CAPE and CAPE-*p*NO_2_ in HT-29 cells and in organs (tumour, heart, liver, spleen and kidney). In our results, the main difference is that CAPE can combine with the glucose acid, while para nitro-benzene alcohol combined with glucose acid after the hydrolysis of CAPE-*p*NO_2_. In CAPE, caffeic acid from CAPE hydrolysis was methylated; however, in CAPE-*p*NO_2_, caffeic acid from CAPE-*p*NO_2_ hydrolysis combined with L (+)-cysteine (maybe from the HT-29 cell line). C_17_H_15_NO_5_ only occurred in the metabolite of CAPE-*p*NO_2_.

On the other hand, the metabolites of CAPE and CAPE-*p*NO_2_ in HT-29 cells were different in the heart, liver, spleen and kidney. CAPE was transformed into C_13_H_17_NO_3_S, C_8_H_11_O_4_P and C_14_H_16_N_2_O_6_ in the heart, liver and kidney, respectively. Compared with CAPE, CAPE-*p*NO_2_ was transformed into 4 metabolites, C_14_H_16_N_2_O_6_, C_8_H_11_O_4_P, C_10_H_14_N_2_O_6_S and C_16_H_11_NO_7_. The differences in the metabolites in cells and organs may be due to the discrepancy of the species of metabolic enzyme and their types. However, there were no metabolites detected in tumours, implying that CAPE and CAPE-*p*NO_2_ were transformed to other compounds in tumours and need to be further studied in the future. Additionally, according to the pharmacokinetic analysis of CAPE and CAPE-*p*NO_2_ in rats, the half-lives (t_1/2_) of these compounds were 4.2 h and 20.9 h, respectively, and the area under concentration-time curve (AUC_all_) values were 1659 and 3239, respectively, indicating that the bioavailability of CAPE-*p*NO_2_ was higher than that of CAPE, and the metabolic processes of CAPE and CAPE-*p*NO_2_ would be different^46^. The most of above metabolites are inactive excrement of CAPE or CAPE-*p*NO_2_, and there are no literatures reported the anti-cancer effect of these metabolites detected in this study, the results of LC-MS/MS provided a basis for further study to explore the compounds to against colon cancer with stronger effect only.

## Conclusion

In this study, para-nitro enhanced the anti-colon cancer activity of CAPE, and the present data showed that CAPE-*p*NO_2_ is more effective than CAPE in inducing colon cancer cell death, apoptosis and cell cycle arrest in the G0/G1 phase by regulating the relative proteins in the P53 pathway and inhibiting tumour growth. Moreover, CAPE-*p*NO_2_ significantly decreased the expression of VEGF. The metabolites of CAPE-*p*NO_2_ were different from those of CAPE in HT-29 cells and organs. This study contributes to further development of the pharmacological activity of CAPE-*p*NO_2_.

## Materials and Methods

### Materials

CAPE and p-nitro CAPE (CAPE-*p*NO_2_) were synthesized as previously described^47^. The chemical structures are shown in Fig. 1A and B. The human colon cancer cell line HT-29 and HCT-116 were purchased from the Cell Bank at the Chinese Academy of Sciences (Beijing, China). Dulbecco’s modified Eagle medium-high glucose (DMEM-H) and foetal bovine serum (FBS) were purchased from Gibco/Invitrogen (Carlsbad, CA, USA). Dimethyl sulfoxide (DMSO), trypsin, streptomycin, penicillin, 3-[4,5-dimethyl-2-thiazolyl]-2,5- diphenyl-2-tetrazolium bromide (MTT), propidium iodide (PI) and Hoechst 33342 were obtained from Sigma Aldrich (St. Louis, MO, USA). P53 inhibitor (Pifithrin-α) was purchased form Selleck (USA). An Annexin V-FITC Apoptosis Detection Kit was purchased from Keygen Biotech Co Ltd. (Nanjing, China). RNase A was acquired from Promega (Madison, WI). Bcl-2, Bax, cleaved-caspase-3, pro-caspase-3, P38, P21^Cip1^, P27^Kip^, P53, c-Myc, CDK2, VEGF and β-actin antibodies were purchased from Proteintech Group Inc. (Wuhan, China). For treatment, CAPE and CAPE-*p*NO_2_ were dissolved in DMSO and diluted to a final concentration with culture medium (DMSO concentration < 0.1%). All chemical reagents were used before the date of expiration.

### Cell Culture

HCT-116 and HT-29 cells were maintained in DMEM-H supplemented with 10% FBS, penicillin (100 U/mL), and streptomycin (100 μg/mL) at 37 °C in a humidified incubator with 5% CO_2_ and 95% air.

### Cell Viability Assay

HT-29 and HCT-116 cells were seeded at a density of 3 × 10^3^ cells per well in 96-well plates and were incubated for 24 h. Next, different concentrations CAPE and CAPE-*p*NO_2_ were administered. For pifithrin-α (PFTα, P53 inhibitor) group, cells were treated with the above concentration of CAPE and CAPE-*p*NO_2_ after pre-treatment with 5 μM PFTα, and then the expression of P53 was detected by Western Bolt. After treatment with CAPE and CAPE-*p*NO_2_ for 48 h, MTT (5 μg/mL) was added to each well for 4 h at 37 °C. The amount of MTT crystals (formazan) dissolved by DMSO was measured using a Plate Reader (Bio Tek) at 570 nm. All experiments were repeated three times, and IC_50_ values were calculated.

### Hoechst 33342 staining

Hoechst 33342 was dissolved in PBS, and the final concentration was 10 μg/mL. HT-29 and HCT-116 cells were treated with different concentrations of CAPE and CAPE-*p*NO_2_ after seeding in 6-well plates (8 × 10^4^ per well). After 48 h, the cells were rinsed three times with PBS, and Hoechst 333342 was added. The cells were then incubated at 37 °C in the dark for 30 min. Thereafter, the dye liquor was removed, and the cells were washed three times with PBS. A fluorescence microscope (Olympus U-RFLT50, Tokyo, Japan) was used for the examination of each well at 6 different fields of view.

### Cell apoptosis and cell cycle analysis by flow cytometry

HT-29 and HCT-116 cells were seeded in 6-well plates at a density of 8 × 10^4^ per well. After 24 h, different concentrations CAPE and CAPE-*p*NO_2_ were added. Next, floating cells (digested by trypsin) were collected after treatment with CAPE and CAPE-*p*NO_2_ for 48 h. Cells were re-suspended in 500 μL of binding buffer after washing twice with PBS. Next, 5 μL of Annexin V-FITC and 5 μL of PI were added, and the cells were incubated for 1 h in the dark. Apoptosis was measured with a FACSCalibur flow cytometer (Keygen Biotech, Co Ltd, Nanjing, China). For cell cycle analysis, after treatment for 48 h, the cells were collected and then fixed with 70% ice-cold ethanol at 4 °C for 24 h. The stationary liquid was removed through centrifugation, and then 100 μL of RNase A and 400 μL of PI were added, followed by incubation at room temperature for 30 min without light. Next, a FACSCalibur flow cytometer (Becton-Dickinson) equipped with a 488-nm argon laser was used, and cell cycle analysis was performed using Cell Quest software and ModFit.

### Western Blotting Analysis

The proteins of HT-29 cells were extracted after treatment with RIPA buffer and 1 mM PMSF (phenylmethylsulfonyl fluoride) on the ice for 30 min, the proteins of tumours were extracted after homogenate with RIPA buffer and 1 mM PMSF at 4 °C for 30 min. Proteins with different molecular weights were separated on 6.0%, 8.0% and 12% SDS-polyacrylamide gels. The proteins on the gel were transferred to a PVDF membrane, which was soaked for 10 seconds with methanol. Next, 5.0% skim milk prepared with PBST (0.1% Tween-20 and 99.9% PBS) was used to block the membrane for 1.5 h. Thereafter, the blots were incubated with primary antibody overnight at 4 °C. The blots were then incubated with the corresponding HRP-linked secondary antibody for 1.5 h after washing with PBST three times. The PVDF membranes were developed by ECL (enhanced chemiluminescence). The protein expression levels were determined by the grey values from the PVDF membrane that were calculated using software Quantity One.

### Analysis of metabolites by LC-MS/MS

HT-29 cells were washed 3 times with PBS and then broken ultrasonically to collect metabolites after treatment for 48 h. Thereafter, centrifugation (1400 × g, 5 min) was applied to separate cell debris and metabolite liquid before injection into the LC-MS/MS instrument. Tissue samples were cut into small sections and then were mixed with liquid (methanol:acetonitrile:acetone:water = 3:3:3:1) to break cells; after homogenates were obtained, the mixed liquor was centrifuged (1600 × g, 10 min), and the supernatant was extracted for LC-MS/MS. LC-30AD (Shimadzu, Japan) conditions were as follows: a Kinetex XB-C18 column (2. 1 mm × 100 mm, 2.6 μm); a mobile phase of acetonitrile and ultrapure water with 0.1% formic acid; a flow rate of 300 μL/min; a column temperature of 30 °C; Triple TOF 4600 (Absciex USA): ionization mode included positive and negative; mass scanning range m/z: 50–300; sheath gas: 55 Pa; auxiliary gas: 55 Pa; curtain gas: 25 Pa; atomization temperature: 600 °C; scanning mode: TOF-MS-Product Ion-IDA; declustering potential: 80 V; CE collision energy 35 eV; CES collision energy: 35 ± 15 eV. Metabolites were analysed by the software Analyst TF 1.6, Peakview (Version 1.2, AB Sciex) and MetabolitePlot (Version 1.5, AB Sciex).

### Xenografts in athymic mice

Four-week-old Male BALB/c nude mice were purchased from the Beijing HFK Bioscience Co Ltd. (Beijing China) and were placed in specific pathogen-free (SPF) conditions. Trypsinized HT-29 cells were injected into the right flanks of athymic mice at a density of 2 × 10^7^/0.1 mL. Water and food for mice were sterilized. When the size of the tumour was approximately 100 mm^3^, the mice were divided into five groups of 10 mice each. These five groups were the control group, CAPE group (10 mg/kg/day), and CAPE-*p*NO_2_ groups (5 mg, 10 mg and 20 mg/kg/day). The tumour volumes were measured every 3 days using a Vernier calliper and were quantitated according to the formula (length × width^2^ × π)/6^48^; the inhibition rate was calculate by the formula inhibition rate (%) = [1 − (the weight of tumours in the experimental/the weight of tumours in control)] × 100%. The tumours were removed and used for detecting other biochemical indexes after CAPE and CAPE-*p*NO_2_ were given for 42 days.

### Haematoxylin and Eosin staining

After treatment, tumours and organs were removed from nude mice and were fixed with 4.0% paraformaldehyde. Paraffin sections were dewaxed to water for different experiments. For HE (haematoxylin and eosin) staining, sections were put into haematoxylin for 8 min and then washed twice before incubating with eosin for 3 min. Finally, the sections were sealed with neutral gum. Nuclei appeared bluish, while the cytoplasm appeared red through the microscope.

### TUNEL staining

Paraffin sections were covered with broken membrane fluid for 20 min. The TUNEL kit reagents were added to the sections for 2 h at 37 °C while maintaining the humidity. Next, endogenous peroxidase was blocked by 3.0% hydrogen peroxide solution prepared in methanol for 15 min. DAB chromogen was used for staining; when positive cells became brown, the staining was stopped by washing with distilled water. Finally, the nuclei were stained with haematoxylin before sealing.

### Immunohistochemistry

Antigen repair buffer (pH 9.0) was added before blocking endogenous peroxidase. The sections were sealed with 3.0% BSA for 30 min. The sections were incubated with the primary antibody (VEGF) overnight at 4 °C. After incubating with PBS three times, the sections were incubated with the corresponding secondary antibody for 50 min. Next, DAB and haematoxylin were used for staining as previously described. The IOD value measured the expression of VEGF.

### Statistical analysis

All data were analysed by SPASS 16.0, the data normality was verified by K-S test, and if the P value was more than 0.05, the data obeyed a normal distribution. The data were presented as the means ± SD for at least three independent experiments. One-way ANOVA and Student’s t-test were performed for statistical analysis. A P value less than 0.05 was considered statistically significant.

### Ethics Statement

This study was carried out in strict accordance with the recommendations in the Guide for the Care and Use of Laboratory Animals from the National Institutes of Health. All animal procedures were approved by the Ethical Committee for Animal Experiments of Southwest University (Permit Number: SYXK 2015-0002). All efforts were made to minimize suffering.

## Electronic supplementary material

supplement
