# Supplementary material for: Anti-colon cancer effect of caffeic acid p-nitro-phenethyl ester in vitro and in vivo and detection of its metabolites
Source: Sci Rep. 2017 Aug 8;7:7599. doi: 10.1038/s41598-017-07953-8 (PMC5548715; doi:10.1038/s41598-017-07953-8)
Supplement: Supplementary file 1 — supplement [file 41598_2017_7953_MOESM1_ESM.pdf]

Anti-colon cancer effect of caffeic acid *p*-nitro-phenethyl ester *in vitro* and *in vivo* and detection of its metabolites

Hao Tang[1], Xiaofang Yao[1], Cong Yao, Xiaoyan Zhao, Hua Zuo, Zhubo Li*

College of Pharmaceutical Sciences, Southwest University, Chongqing, 400716

Affiliation

College of Pharmaceutical Sciences, Southwest University, Chongqing, China

*** Corresponding author**

Prof. Dr. Zhubo Li

College of Pharmaceutical Sciences, Southwest University

No. 2, Tiansheng Road, Beibei, Chongqing 400716, People's Republic of China

Phone: + 862368251225 Fax: + 862368251225

E-mail: lizhubo2004@163.com

[1] Authors made equal contribution to this study.

CAPE

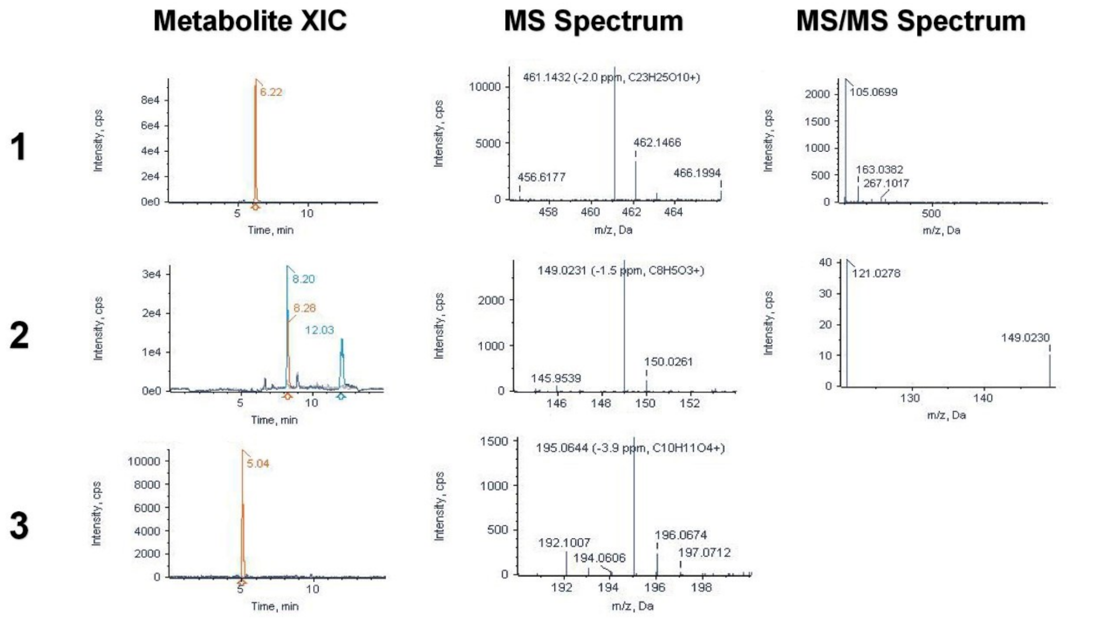

CAPE-pNO_2

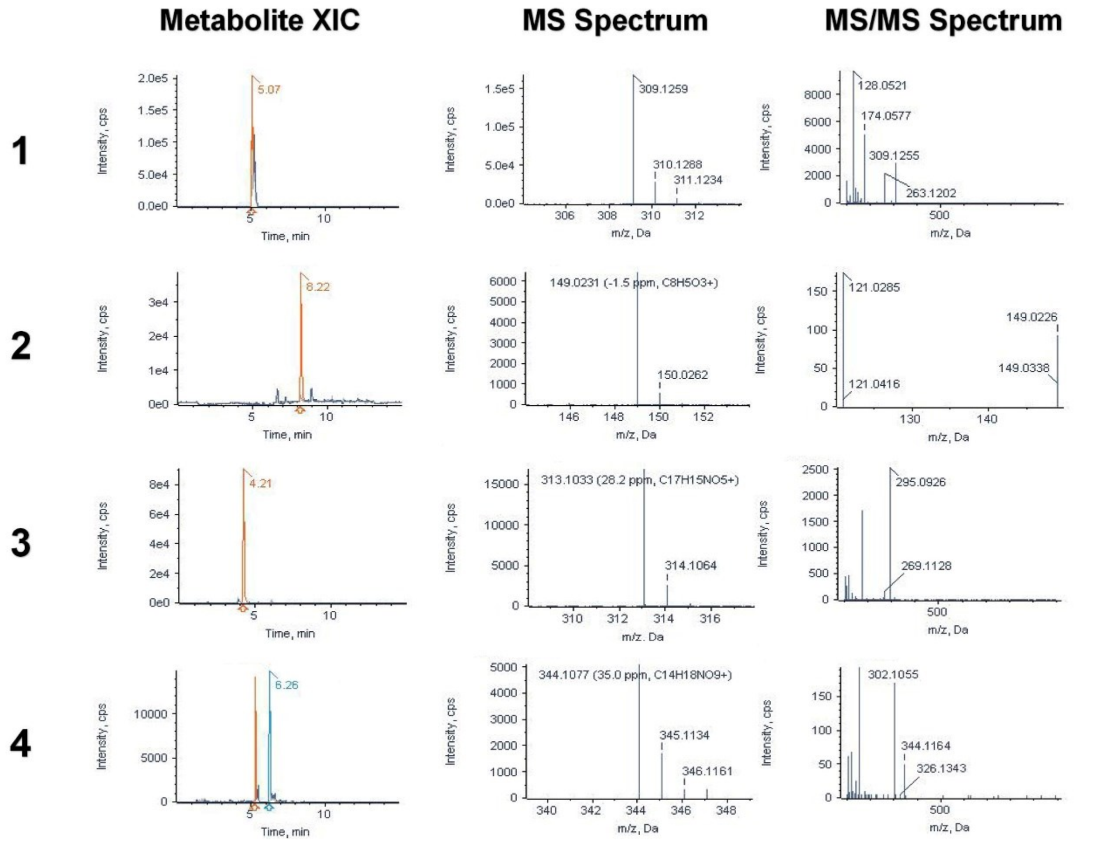

Figure S1. Mass spectrum of metabolites of CAPE and CAPE-*p*NO_2. (A) The mass spectrum of metabolites of CAPE, and the "1, 2, 3" were correspond to the "1, 2, 3" in **Figure 6A**. **(B)** The mass spectrum of metabolites of CAPE-*p*NO_2, and the "1, 2, 3, 4" were correspond to the "1, 2, 3, 4" in **Figure 6B.**

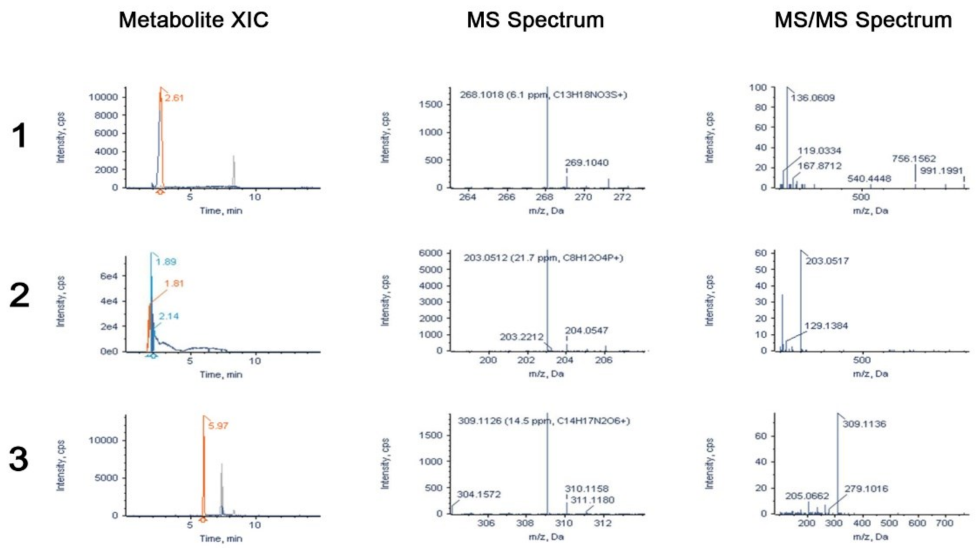

Figure S2. Mass spectrum of metabolites of CAPE. The mass spectrum of metabolites of CAPE, and the “1, 2, 3” were correspond to the “1, 2, 3” in **Figure 7A**.

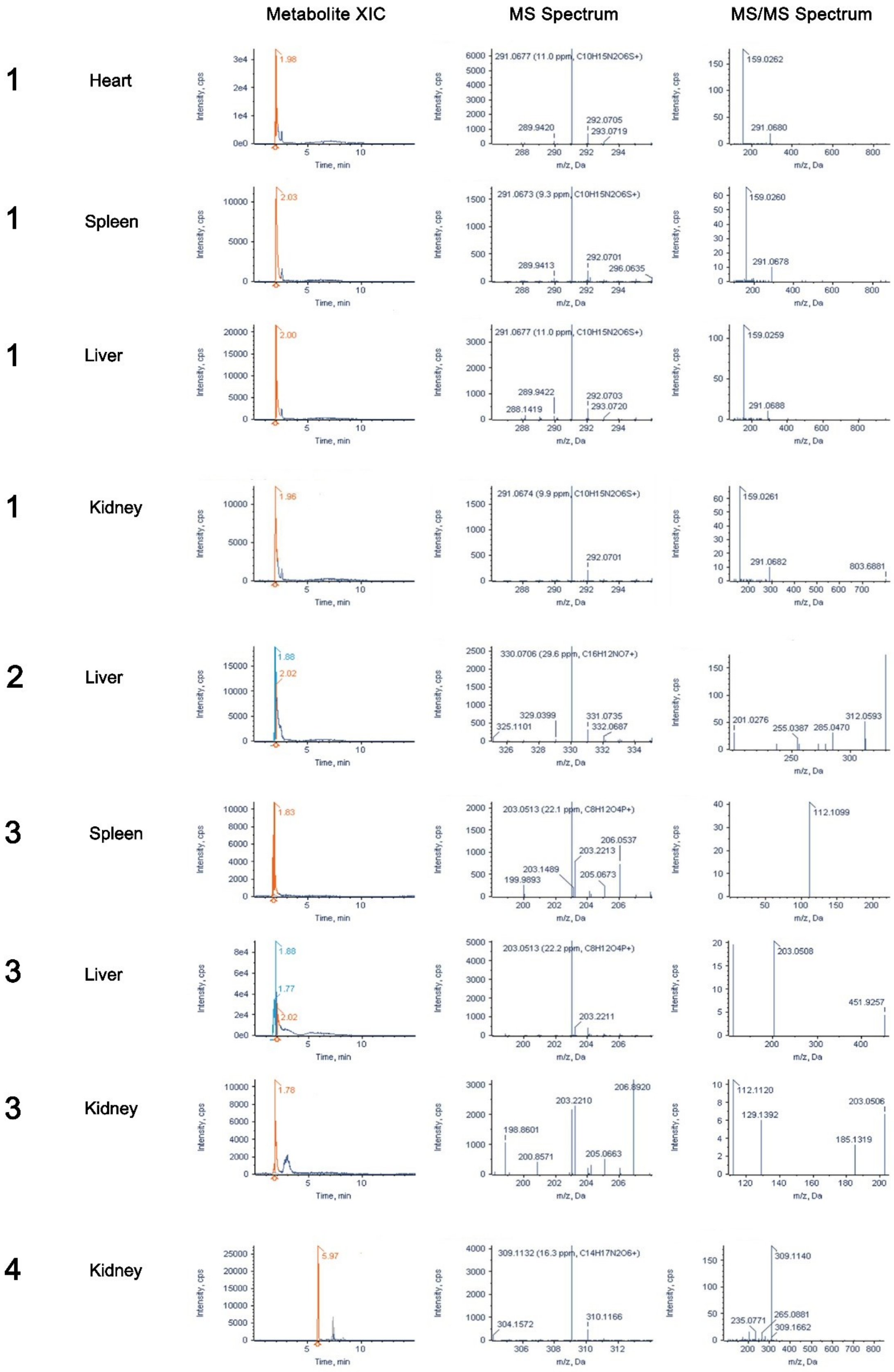

Figure S3. Mass spectrum of metabolites of CAPE-*p*NO$_2$. The mass spectrum of metabolites of CAPE, and the “1, 2, 3, 4” were correspond to the “1, 2, 3, 4” in **Figure 7B**.

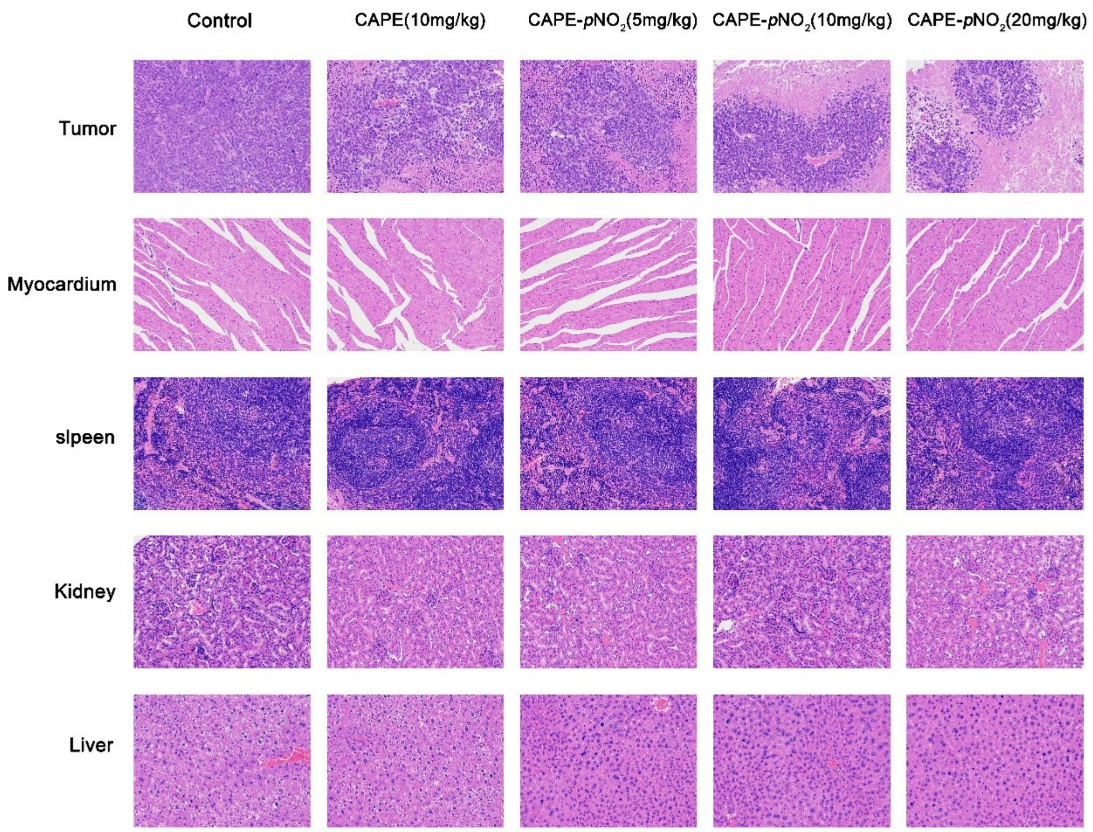

Figure S4. HE staining. Heart, liver, kidney and spleen Paraffin sections of heart, liver, kidney and spleen were stained by hematoxylin and eosin (HE), and there almost no observable morphological changes after treatment with CAPE and CAPE-*p*NO_2.
